# 

**Published:** 1930

**Authors:** 


### CONTENTS.

| | Page |
|---|---|
| The Duty of Medicine to the Race. By C. Ferrier Walters, F.R.C.S. | 1 |
| Hospitals—Voluntary or Self-Supporting? By R. J. A. Berry, M.D., F.R.C.S., F.R.S. | 19 |
| The Infective Factor in Thrombosis and Embolism. By Ambrose W. Owen, M.D. | 29 |
| Reviews of Books | 69 |
| Editorial Notes | 76 |
| Meetings of Societies | 78 |
| The Medical Library of the University of Bristol | 84 |
| Local Medical Notes | 87 |

